# 

**Published:** 1904-11

**Authors:** 


					ESTABLISHED IN 1839
THE OLDEST DENTAL MAGAZINE IN THE WORLD
INDEPENDENT PUBLISHED BY DENTISTS FOP DENTISTS
VOLUME XXXV
NUMBER II
THIRD SERIES
NOV.
1904
Editor :
VVm. Gird Beeckoft, D D. S , Madison, Wis.
Associate Editors:
Albert H. Ketcham, D D. S.. Den-er, Colo
Emoey A. Bkyant, D. D S., Washington, D. C.
Ueo. S Martin, D D. S., L. D. S Toronto Junction,
Ontario.
Puhlisheu on the 10th day of each month.
Subscription Price, $1.00 per year.
Foreign Countries, $1.50 pe year.
Address all cominuuic itions, both business and editorial
to the editor
Entered an Second Class Matter, Madison,
Wis.
The eighth annual meeting of the
Southern: Branch of the National Dental
Association will be held Feb. 21-23, 1905,
at Memphis, Tenn.
J. At Gorman, Cor. Sec'jj
Asheville, K C.
The regular semi-annual meeting of the
Colorado Slate Bbard of Dental Exam-
iners will be held in Denver, December
6th, 7th and 8th, 1904.
The examination will be both theoreti-
cal and practical and applicants for the
examination must be prepared to do such
practical work as is required.
All applications must* be filed before
December 6th.
M. S>. Fbaser, Sec'v,
407 Mack Block,
Denver, Colo.

				

